# 857. Hospital-onset Bacteremia in Burn Patients: Prevalence and Characteristics in a Regional Burn Center

**DOI:** 10.1093/jbcr/irag033.327

**Published:** 2026-04-06

**Authors:** Alessandra E Cabrera, Cristian Cazac, Julie A Caffrey, Geeta Sood

**Affiliations:** Johns Hopkins Adult Burn Center, Baltimore, MD; Johns Hopkins University School of Medicine, Baltimore, MD; Johns Hopkins University School of Medicine, Baltimore, MD; Johns Hopkins University, Baltimore, MD

## Abstract

**Introduction:**

Burn patients are more likely to develop bacteremia due to disruptions of the skin barrier, prolonged use of central lines, and the need for multiple procedures. Hospital-onset bacteremia, defined as bacteremia occurring after 3 days of admission, has been proposed as a quality measure for value-based purchasing programs. We sought to evaluate the prevalence and characteristics of bacteremic episodes in our high-volume regional burn center.

**Methods:**

Patients admitted to a high volume burn center between September 2018 - December 2024 were identified through the burn registry. Patients admitted with severe skin conditions such as toxic epidermal necrolysis were also included. Electronic medical records were reviewed to identify cases of bacteremia. Bacteremia was defined as a positive blood culture obtained for clinical indications using standard clinical microbiological methods. Multiple blood cultures obtained on the same day or multiple organisms isolated from the same blood cultures accounted for a single episode of bacteremia. Microbiological, demographic, and coding data were extracted from the patients’ medical records. Procedures were identified through coding data. Muscle and skin procedures were identified using CCS codes 159, 160, 163, 164, 168 -175.

**Results:**

There were 1612 initial admissions for burn injuries for 1590 patients between September 2018 - December 2024 at our regional burn center. 1085 patients had 0%-10% total body surface area (TBSA) burns, 260 patients had 10-39% TBSA burns, and 56 patients had >40% TBSA burns. TBSA data was not available for 211 patients. 89 patients (5.6% of all patients) developed 202 episodes of bacteremia during their hospitalization. 57 patients developed one episode of bacteremia. Three patients developed multiple episodes of bacteremia each having had 12, 14, and 19 episodes of bacteremia. These patients had TENS, 36% TBSA burns, and 42.5% TBSA burns respectively. The most common organisms isolated were *Pseudomonas Aeruginosa*, methicillin-resistant *Staphylococcus Aureus*, collective coagulase-negative Staphylococcus species, and methicillin-sensitive *Staphylococcus Aureus*. 72 of the 202 episodes of bacteremia occurred within 2 days of surgery.

**Conclusions:**

Hospital-onset bacteremia occurred in 5.6% of burn patients, most commonly perioperatively, and most commonly due to *Pseudomonas Aeruginosa* or *Staphylococcus Aureus*. These complications can often prolong hospitalization and resource use. These findings highlight the need for targeted prevention, perioperative infection control, and risk-adjusted benchmarking in burn care.

**Applicability of Research to Practice:**

The prevalence of hospital-onset bacteremia was 5.6% in burn and severe skin injury patients. This finding reinforces the need for targeted empiric therapy, enhanced infection prevention, and risk-adjusted benchmarking in burn care.

**Funding for the study:**

N/A.

